# Development of the New Fluoride Ion-Selective Electrode Modified with Fe_x_O_y_ Nanoparticles

**DOI:** 10.3390/molecules25215213

**Published:** 2020-11-09

**Authors:** Josip Radić, Marija Bralić, Mitja Kolar, Boštjan Genorio, Ante Prkić, Ivana Mitar

**Affiliations:** 1 Department of Environmental Chemistry, Faculty of Chemistry and Technology, R. Boškovića 35, 21000 Split, Croatia; jradic@ktf-split.hr; 2 Department of Chemistry and Biochemistry, Faculty of Chemistry and Chemical Technology, Večna pot 113, 1000 Ljubljana, Slovenia; Mitja.Kolar@fkkt.uni-lj.si; 3 Department of Chemical Engineering and Technical Safety, Faculty of Chemistry and Chemical, Technology, Večna pot 113, 1000 Ljubljana, Slovenia; Bostjan.Genorio@fkkt.uni-lj.si; 4 Department of Analytical Chemistry, Faculty of Chemistry and Technology, R. Boškovića 35, 21000 Split, Croatia; prkic@ktf-split.hr; 5 University of Split, Faculty of Science, Department of Chemistry, R. Boškovića 33, 21000 Split, Croatia; imitar@pmfst.hr

**Keywords:** ion-selective electrode, fluoride, metal nanoparticles, internal contact, elemental analysis

## Abstract

A new modified ion-selective electrode with membranes of LaF_3_ single crystals with different internal contacts (solid steel or electrolyte) and with Fe_x_O_y_ nanoparticles as loading was developed. The best response characteristic with linear potential change was found in the fluoride concentration range from 10^−1^ to 3.98 × 10^−7^ M. The detection limit for the electrolyte contact was determined at 7.41 × 10^−8^ M with a regression coefficient of 0.9932, while the regression coefficient for the solid contact was 0.9969. The potential change per concentration decade ranged from 50.3 to 62.4 mV, depending on whether the contact was solid or electrolytic. The prepared modified electrode has a long lifetime, as well as the possibility of application in different positions (solid contact), and it can also be used for the determination of iron ions. The electrode characterization was performed with scanning electron microscopy and elemental analysis with the technique of electron-dispersive X-ray spectroscopy.

## 1. Introduction

Detection of fluoride and its complexes plays an important role in understanding the benefits, as well as the potential toxicity, of fluoride natural sources [1]. The fluoride ion-selective electrode (FISE) with LaF_3_ membrane is probably the most widely used ion-selective electrode (ISE) for practical measurements [2,3,4]. The electrode was described first by Frant and Ross [5]. Commercially available models, including the Orion Model 94-09, are constructed in the conventional ISE way, with the membrane arranged symmetrically between two solutions. Often, the life of the electrode is shortened because a reference electrode has lost contact with the membrane.

In many applications, such as online process analysis and clinical analysis, it is advantageous to replace the internal reference solution with a fixed contact. Solid contacts allow the construction of electrodes that can withstand high temperatures and pressures (e.g., autoclaving). To achieve the desired electrode quality in terms of sensitivity, response time, and stability, it is essential that the contact materials are in thermodynamic equilibrium at zero current. If equilibrium is not reached, potential instability and long-term potential drift can generally be expected. These effects depend on the speed of the relaxation processes, for example, at the blocked interface between an electronic and an ionic conductor [6].

Previously, internal contacts, based on the use of Ag_2_S, between the LaF_3_ membrane and a stainless-steel disc of the multipurpose solid-state electrode body [7], the redox reference system [8], and the Cu(II) ISE [9] were described. Bralic et al [6] described FISE with a simple solid contact using a laboratory version of the ISE body and a stainless-steel disc. In this instrument, the LaF_3_ membrane was built into a multipurpose electrode body, and contact with the instrument was made with a stainless-steel disc and coaxial cable. An ion-selective solid-state fluoride electrode was developed consisting of 70% Ag_2_S, 10% Cu_2_S, and 20% CaF_2_ [10].

The production of a novel FISE is necessary for several reasons; fluoride is important for trade and technology, its small quantities are vital for the human body although larger quantities are toxic, and its determination using previous techniques is difficult.

In recent years, much attention has been paid to the study of different types of cheap and efficient materials, such as different clays, for the removal of fluoride from the environment, especially from drinking and wastewater. Recently, fluoride ions have been investigated by adsorption on synthesized Fe_2_O_3_ nanoparticles [11,12,13].

Nanomaterials play an important role in the production of chemo- and biosensors, especially due to their unique physical and chemical properties, such as high surface/volume ratio, good conductivity, excellent electrocatalytic activity, and high mechanical strength. In recent years, nanomaterials have been gradually introduced into potentiometric sensors precisely because of their properties. For example, due to their exceptional electrical properties and good hydrophobicity, nanomaterials are suitable for ISE use in the solid state, since they can be dispersed directly in ion-selective membranes [14].

The manufacture of a gold-based ion sensor coated with Fe_2_O_3_ nanoparticles for the determination of fluoride is also described [15].

In reviewing the literature, we have not found recent work describing an FISE with metal nanoparticles. This paper describes an FISE with LaF_3_ membranes of different thickness and with different Eu ratios, to which iron oxide nanoparticles (Fe_x_O_y_ NPs) were loaded. The purpose of Fe_x_O_y_ NP loading was to improve the response characteristics of the prepared electrode in relation to the commercial FISE. The LaF_3_ membrane was mounted in a multipurpose electrode body, and contact with the instrument was made with a stainless-steel disc and coaxial cable or an internal electrolyte contact and an Ag/AgCl internal electrode. The electrode surfaces were recorded using scanning electron microscopy (SEM) and an elemental analysis was performed using electron-dispersive X-ray spectroscopy (EDS).

## 2. Results and Discussion

### 2.1. Potentiometric Measurements

The potential response of the FISE was measured using a two-electrode system. The solution was stirred and monitored throughout successive additions of known amounts of sodium fluoride. The FISE response to fluoride ion concentration is given by the Nernst equation.

(1) E=E′+S×pF

where E, E′, and S denote the cell potential after addition of sodium fluoride, a conditional standard cell potential, and experimental slope, respectively.

Electrodes with membranes that were not treated with Fe_x_O_y_ NPs showed linearity in the range within two concentration decades with a slope in the range from 10.4 to 33.9 mV for internal solid contact or in the range from 21.2 to 47.5 mV when the internal contact was electrolyte (Figures S1 and S2, Supplementary Materials; Table 1).

**Figure 1:**
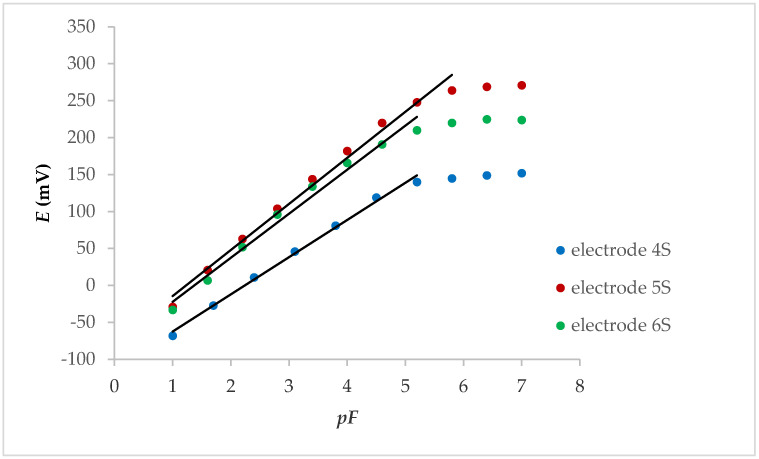
Potentiometric response of internal solid contact LaF_3_ electrodes with Fe_x_O_y_ NPs.

After preliminary measurements (without Fe_x_O_y_ NPs), each of the electrodes mentioned above was tested after the Fe_x_O_y_ NP loading on membranes. All three tested electrodes showed a linear potential change that was lower than the fluoride concentration of 1.00 × 10^−5^ M, while the potential changes per concentration decade at the internal solid contact were between 50.3 and 62.4 mV (Figure 1).

**Figure 2:**
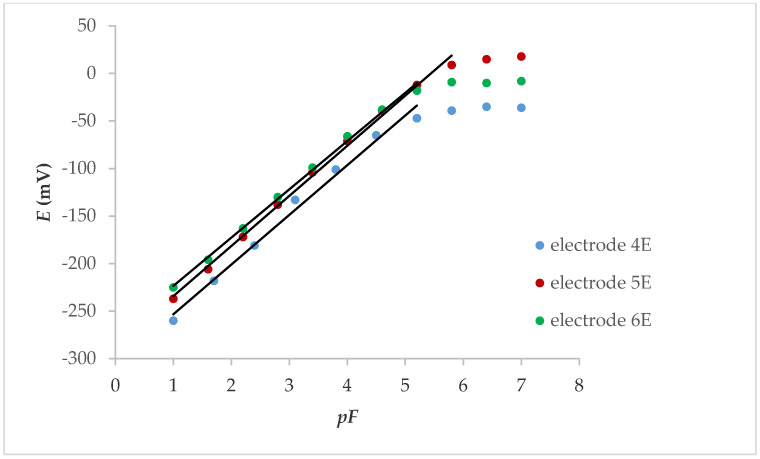
Potentiometric response of internal electrolyte contact LaF_3_ electrodes with Fe_x_O_y_ NPs.

If the internal contact was an electrolyte (Figure 2), the linear response was also in the concentration range below 1.00 × 10^−5^ M, while the slope was between 50.8 and 52.7 mV, depending on whether the electrode was conditioned (24 h in 0.001 M KNO_3_ solution) or not. The response characteristics of the electrodes are shown in Table 2.

After the measurements described above, the Fe_x_O_y_ NPs were washed off the membrane surface so that the membranes were left in 1 M nitric acid solution for 24 h. The response of the electrodes was tested, and it was observed that, after washing the Fe_x_O_y_ NPs out from the surface, the electrodes showed an even wider linear range. The potential change per concentration decade was in the range of 52.9 to 57.3 mV for solid-state contact, while it was in the range of 44.1 to 54.3 mV for electrolyte contact (Figures S3 and S4, Supplementary Materials; Table 3).

Furthemore, the response of the electrode 7E to fluoride ions was compared with the response of the commercial electrode (Figure S5, Supplementary Materials).

#### 2.1.1. pH Effect on the Electrode Response

Hydroxide ions are known to have great influence on fluoride determination by electrode with an LaF_3_ membrane. The penetration of OH^−^ ions into the LaF_3_ crystal lattice plays an important role. The consequence is the release of F^−^ ions from the lattice, their diffusion into solution, and a change in potential.

(2) $$F_{\left(\mathrm{lattice}\right)}^{-}+{\mathrm{OH}}_{\left(\mathrm{solution}\right)}^{-}⇄F_{\left(\mathrm{solution}\right)}^{-}+{\mathrm{OH}}_{\left(\mathrm{lattice}\right)}^{-}$$

**Figure 3:**
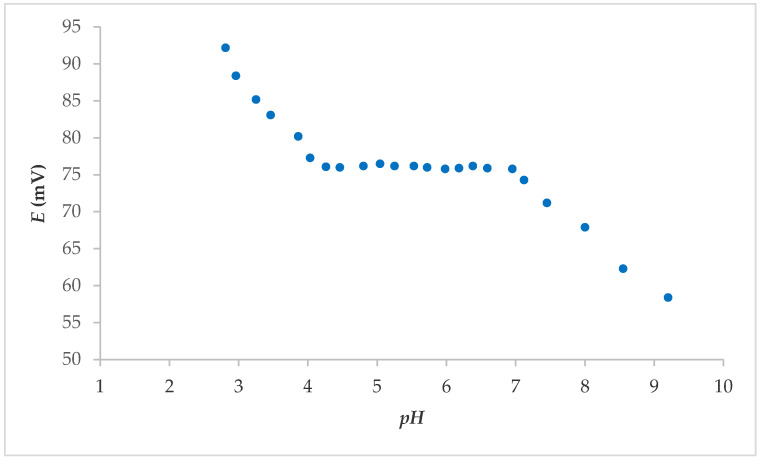
pH effect on the electrode 7S response in F^−^ solution (concentration: 1.00 × 10^−3^ M), after washing Fe_x_O_y_ NPs from surface.

From the above, it was concluded that electrodes 7S and 7E showed the best properties overall; thus, it was further tested. The effect of the pH was determined by studying the fabricated electrode in solutions with an F^−^ concentration of 1.00 × 10^−3^ M. The pH value was varied from 3 to 9 with the addition of NaOH. The potential change was a function of the pH value. The pH influence of electrode 7S is shown in Figure 3. As shown, the reaction of the sensors in the range 4–7 was independent of the pH influence. No visible interference from H_3_O^+^ or OH^−^ ions was observed in this pH range. The pronounced influence of pH on the FISE is usually in the pH range below 4 and above 9 [16].

#### 2.1.2. Response Time and Electrode Characteristics

**Figure 4:**
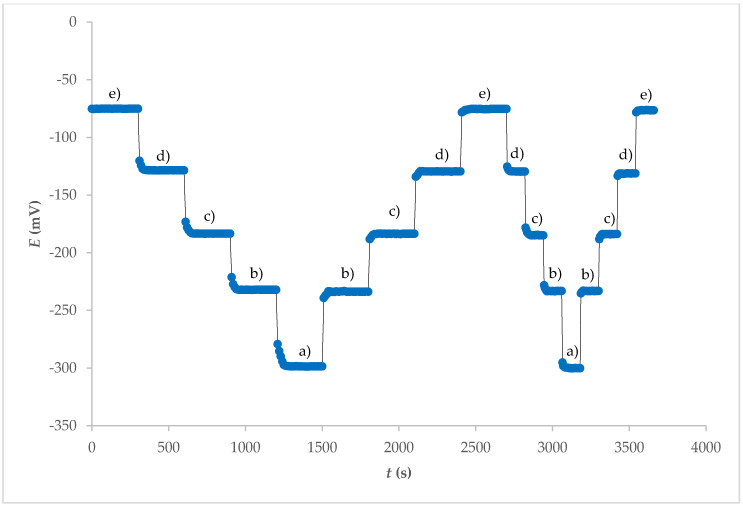
Dynamic response and reproducibility of electrode 7E for different concentrations of F^−^ at pH = 5 after washing Fe_x_O_y_ NPs out from surface: (**a**) 1 × 10^−1^ M; (**b**) 1 × 10^−2^ M; (**c**) 1 × 10^−3^ M; (**d**) 1 × 10^−4^ M; (**e**) 1 × 10^−5^ M.

The response time of an ISE is also an important factor for any analytical application. Experimental conditions such as stirring, ionic strength, and composition of the test solution, as well as the concentration and composition of the solution to which the electrode was exposed, can have an influence on the experimental response time of a sensor. Before the experimental measurements are carried out, any previous use or preconditioning of the electrode and the test temperature can also have an influence on the response time [17]. The potential–time response curve of the electrode obtained from the internal electrolyte contact for different concentration ranges of F^−^ ions is shown in Figure 4. The stationary potential was reached within 1 min. A similar response time, but in a smaller concentration range, was observed with fluoride ion sensors based on a crystal cadmium (II) Schiff base complex [18].

After the removal of Fe_x_O_y_ NPs from the membrane surface, the response properties were improved for all electrodes tested. It is possible that iron from the oxide reacted with the F^−^ ions (reaction 2) from the solution to form an FeF_2_^+^ or FeF^2+^ complex [19], which influenced the electrode reaction or contributed to the improved conductivity, since Fe was embedded in the membrane itself, as shown by the elemental analysis of the membrane (Table 4) and the changes observed on the membrane of the electrode surface.

(3) $${\mathrm{Fe}}^{3+}+nF^{-}⇆{\mathrm{FeF}}_{n}^{\left(3-n\right)+}$$

Furthermore, it is obvious that the thickness of the membrane and the ratio of Eu influence the reaction properties. Specifically, the 1 mm thick membrane with 1% Eu showed the best response characteristics.

Compared to some of the FISEs described above [10], the electrode in this study showed a lower detection limit and easier replacement of the internal contacts. Moreover, the electrode described in this paper was much easier to prepare than the electrodes in the article mentioned. 

The described electrode showed a better response than the electrode based on the crystal cadmium (II) Schiff base complex [18] and a similar response to a gold-based electrode coated with β-Fe_2_O_3_ [15], but the regression coefficient was better in this work.

#### 2.1.3. Lifetime of Electrode

**Figure 5:**
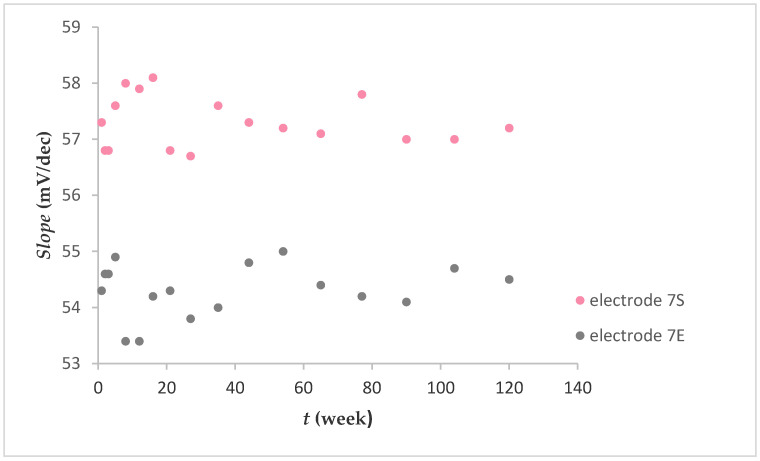
Fluoride response over 120 weeks (after washing Fe_x_O_y_ NPs out).

Figure 5 shows the fluoride sensitivity expressed as mV per decade change in concentration (mV/dec) over a 120 week period. Electrodes 7S and 7E exhibited fluoride sensitivity with an average value of 54.3 ± 0.5 mV/dec and 57.3 ± 0.4 mV/dec, respectively. There were no noted losses in sensitivity. Generally, after preparing the electrodes and their use, they were stored in air. It was found that a prolonged dry storage of electrodes had no measurable effect on their responses.

In contrast to the graphene-based FISE [20], where the lifetime of the electrode was limited, the lifetime of the electrode described in this paper (Figure 5) was almost unlimited (up to mechanical cracking).

#### 2.1.4. Iron Ion Response Characteristics and Influence of the Interfering Ions 

As it is known that fluorine forms stable complexes in water with a series of metal ions (most commonly with Al^3+^, Be^2+^, and Fe^3+^ ions), an electrode prepared in this way can be used to determine them [21].

**Figure 6:**
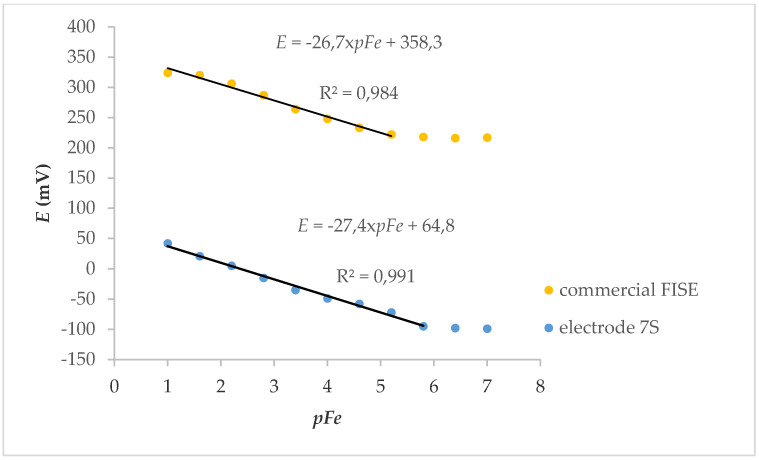
Calibration curves for determination of Fe^3+^ with commercial and 7S fluoride ion-selective electrode (FISE).

Electrode 7S was applied to the determination of iron ions, and the results are shown in Figure 6. A wider linear range with respect to the commercial electrode (and, consequently, a lower limit of detection) was observed.

**Figure 7:**
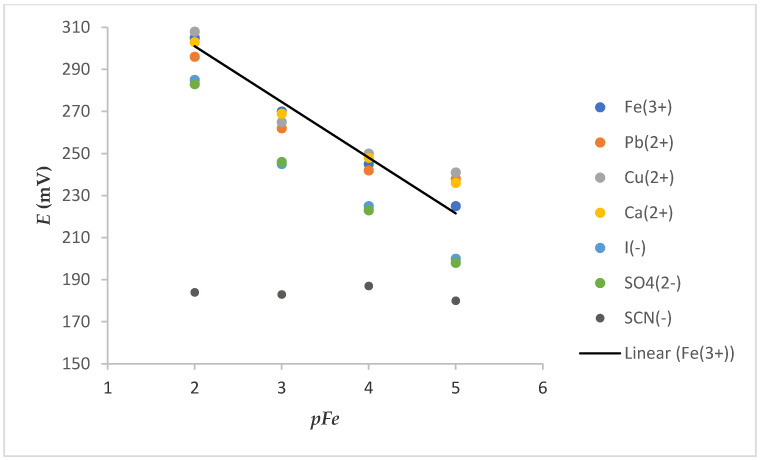
Dependence of E vs. pFe on electrode 7S (after washing Fe_x_O_y_ NPs out) in the presence of a constant concentration of various cations or anions (c = 0.01 M).

The selectivity of the ISE is one of its most important characteristics. It indicates the specificity of the sensor toward the target ion in the presence of interfering components. Slightly parallel shifts of calibration curves (Figure 7) were obtained in the presence of the tested cation (0.01 M) solution. This shift, which is more discernible at a low concentration of Fe^3+^, can be attributed to the change in ionic strength in the solution because of a high concentration of interfering cations. Some deviations were also observed in the presence of tested anions. These deviations were manifested as a decrease in the slope and could be explained by oxidoreduction reactions in the case of I^−^. The impossibility of iron determination in the presence of SCN^−^ was probably due to strong complexation of Fe^3+^ with SCN^−^ (K_Fe(SCN)3_ = 10^9^).

### 2.2. Membrane Characterization Using SEM

**Figure 8:**
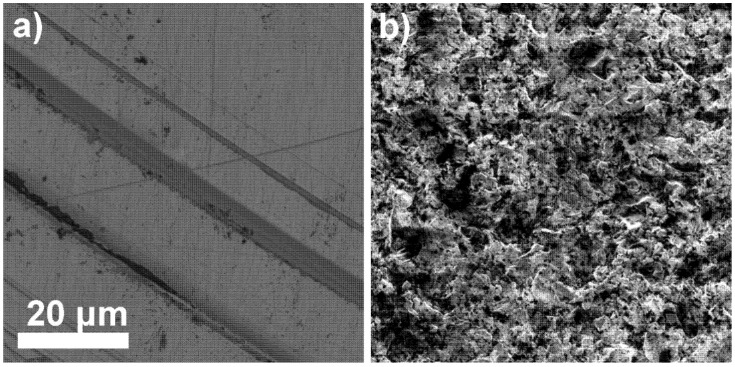
LaF_3_ single-crystal membrane (with diameter of 8.0 mm and thickness of 1.0 mm, doped with 1.0% Eu) surface at 10,000× magnification (**a**) before Fe_x_O_y_ NPs loading and (**b**) after leaching out some Fe_x_O_y_ NPs.

The LaF_3_ single-crystal membrane with diameter of 8.0 mm and thickness of 1.0 mm, doped with 1.0% Eu was morphologically analyzed (before the Fe_x_O_y_ NP loading onto the membrane surface and after membrane acid treatment) using SEM at 3 kV. The membrane surface was smooth before the Fe_x_O_y_ NP loading; however, tiny grooves are visible in Figure 8a, resulting from the polishing procedure. On the other hand, significant changes were observed on the Fe_x_O_y_ NP surface-modified membrane after 24 h treatment in 1 M nitric acid solution (Figure 8b). The surface morphology in Figure 8a,b is noticeably different. The first surface is smooth with no porosity, while the second is rough with high surface area and visible open macropores. The reason for such a difference in surface morphology was the leaching out of some Fe_x_O_y_ NPs from the electrode composite (LaF_3_/Fe_x_O_y_ NPs).

### 2.3. Membrane Elemental Analysis Using EDS

**Figure 9:**
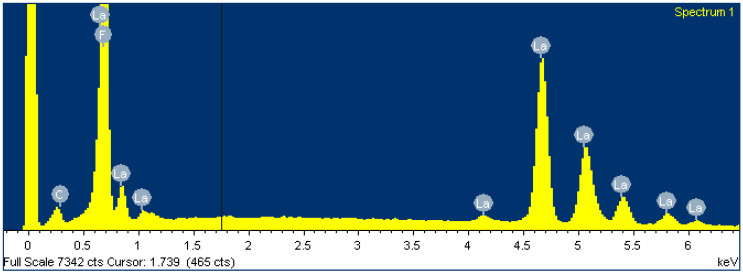
Spectrum of LaF_3_ single-crystal membrane (with diameter of 8.0 mm and thickness of 1.0 mm, doped with 1.0% Eu) before Fe_x_O_y_ NP loading.

To gain insight into the chemical composition of the membrane before and after loading the Fe_x_O_y_ NPs, an EDS analysis (within SEM) was performed. It is clear from Table 4 and Figure 9 that the membrane is chemically composed of fluorine and lanthanum in an atomic ratio of 1 to 3 and a small amount of adventitious carbon. However, this is to be expected and is consistent with the primary LaF_3_ composition of the membrane.

**Figure 10:**
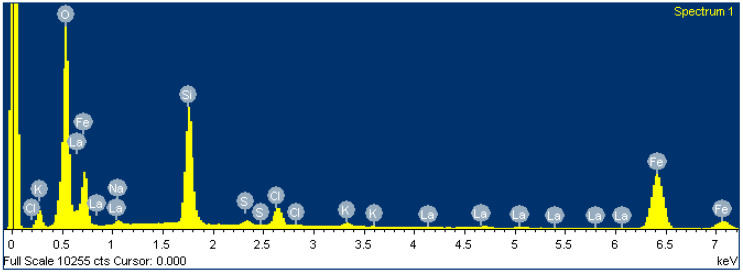
Spectrum of LaF_3_ single-crystal membrane (with diameter of 8.0 mm and thickness of 1.0 mm, doped with 1.0% Eu) after Fe_x_O_y_ NP loading.

After Fe_x_O_y_ NP membrane loading, the EDS composition showed relatively thick Fe_x_O_y_ NP layers. The results from EDS (Table 5 and Figure 10) are consistent with the expected chemical composition of Fe_x_O_y_. Since the substrate LaF_3_ was not detected by EDS, the Fe_x_O_y_ NPs deposits on the membrane were several µm thick. In addition, some impurities were also detected in low concentrations, which were residues from Fe_x_O_y_ NPs synthesis [22,23] that could not be removed.

## 3. Materials and Methods

### 3.1. Apparatus

The Millivoltmeter Mettler-Toledo GmbH Seven Easy was used to measure the potential of the FISE against an Ag/AgCl single-junction reference electrode in a reaction vessel at 25 °C. A lightly constructed multipurpose electrode body [8,9] was used for the assembly of the LaF_3_ membrane. The following LaF_3_ membranes were used for the measurement: with diameter of 8.0 mm and thickness of 1.0 mm, doped with 1.0% Eu (membrane 1); with diameter of 8.0 mm and thickness of 1.5 mm, doped with 0.3% Eu (membrane 2); with diameter of 8.0 mm and thickness of 5.0 mm, doped with 1.0% Eu (membrane 3). All three were manufactured by Crystran Ltd, United Kingdom. The internal contact between the Ag/AgCl reference electrode and LaF_3_ membrane was electrolytic or solid. An Orion fluoride ion-selective electrode Model 94-09 SC was used as a commercial electrode.

Characterization of the membrane surface and microstructure was performed with the Zeiss ULTRA plus (SEM) scanning field-emission electron microscope (Jena, Germany). Furthermore, an elemental analysis of the membranes inside SEM was performed with an EDS Oxford X-Max SDD detector (Oxford, United Kingdom) with a working area of 50 mm^2^, which was processed with INCA 4.14 5 software (Oxford Instruments, Oxford, United Kingdom). The SEM images were taken at 3 kV, while the EDS analysis was performed at 20 kV.

### 3.2. Reagents

All chemicals used were of analytical grade and were used as received without further purification. Sodium fluoride, hydrochloride acid, and silver nitrate were supplied by Sigma-Aldrich, Schnelldorf, Germany. Anhydrous sodium acetate was purchased from Gram-mol, Zagreb, Croatia. Glacial acetic acid, potassium nitrate, potassium chloride, potassium rhodanide, potassium sulfate, potassium iodide, calcium nitrate tetrahydrate, lead(II) nitrate, copper(II) nitrate trihydrate, and iron(III) nitrate nonahydrate were purchased from Kemika, Zagreb, Croatia. The solutions were prepared with double-distilled water.

Standard sodium fluoride solution (0.1000 M) was prepared in a polypropylene calibrated flask from dried (110 °C) sodium fluoride. The diluted standard solution fluoride was prepared by mixing the sodium standard solution fluoride with 0.10 M KNO_3_ and acetate buffer using propylene flasks and pipettes. The stock Fe^3^ solution (0.01 M) was prepared by weighing and dissolving an appropriate amount of Fe(NO_3_)_3_ in 0.10 M KNO_3_ and acetate buffer. Fe^3+^ was titrated using a standardized 0.01 M ethylenediaminetetraacetic acid (EDTA) solution. Other solutions of iron were prepared from the stock solution by dilution with 0.1 M KNO_3_ and acetate buffer. Solutions of interfering ions were prepared in the same way as the Fe^3+^ solution. Acetate buffer, pH 5, was prepared by diluting glacial acetic acid (6.5 mL) and sodium acetate (16.3 g) in distilled water using a 1000 mL volumetric flask.

The electrode inner electrolyte solution was prepared by mixing 10 mL of saturated KCl, 1 mL of concentrated HCl, and one or two drops of 0.10 M AgNO_3_. The preparation and characterization of Fe_x_O_y_ NPs have already been described in the literature [22,23].

### 3.3. Potentiometric Measurements

To measure the potential reaction, 50.0 mL of 0.1000 M NaF, prepared in 0.10 M KNO_3_ in acetate buffer solution, was added to the reaction vessel at 25 °C. The potential response of the FISE was measured by serial dilution (to 10^−7^ M) of the cell solution. During the measurements, the solution was stirred with a polytetrafluoroethylene (PTFE)-coated magnetic rod. The potential–time behavior of the electrode was measured using a regular analysis set-up and recorded on a computer.

### 3.4. Elemental Analysis

Membranes were adhered to the aluminum SEM holder with conductive carbon tape and introduced to the SEM. The membranes were then analyzed using an Oxford X-Max SDD detector (calibrated by Co-Standard, Haarlem, The Netherlands) inside the SEM at 20 kV using point analysis.

## 4. Conclusions

For the first time, an FISE with an LaF_3_ membrane coated with Fe_x_O_y_ NPs was prepared. The membranes of LaF_3_ single crystals were of different thicknesses and had different Eu ratios. The Eu ratio and the membrane thickness influenced the response of the electrodes. Without Fe_x_O_y_ NP loading, the electrodes showed non-Nernstian behavior. After treatment of the electrode with Fe_x_O_y_ NPs, the potential change per concentration decade increased and ranged between 44.1 and 62.4 mV. A detection fluoride limit of 7.41 × 10^−8^ M was calculated. Loss in electrode sensitivity on fluoride determination was not observed over 2 years.

Iron ions are able to form complexes with fluoride ions; thus, the prepared electrode is selective for iron ions. A detection limit for iron below a concentration of 10^−5^ M was observed. No significant influence of cations as an interfering species was observed, while the pronounced interfering species constituted SCN^−^ anions. An elemental analysis after Fe_x_O_y_ NP loading showed that they were mainly present in a thin film on the membrane surface. The advantage of this electrode is preparation simplicity, as well as a solid-state contact, which allows the electrode to be used in different positions and at higher temperatures.

## Figures and Tables

**Table 1:** The response characteristics of electrodes without Fe_x_O_y_ nanoparticles (NPs).

Electrode	Internal Contact	Linear Response (mol/L)	Limit of Detection (mol/L) (LOD)	Slope (mV/dec) ± SD ****	*R* ^2^
1S *	solid	3.16 × 10^−5^	5.89 × 10^−5^	33.9 ± 2.4	0.9390
1E *	electrolyte	3.16 × 10^−5^	3.09 × 10^−5^	35.9 ± 2.0	0.9615
2S **	solid	3.16 × 10^−5^	2.69 × 10^−5^	24.3 ± 1.8	0.9661
2E **	electrolyte	3.16 × 10^−5^	2.51 × 10^−5^	21.2 ± 1.7	0.9683
3S ***	solid	3.16 × 10^−5^	2.63 × 10^−5^	10.4 ± 0.9	0.9668
3E ***	electrolyte	6.31 × 10^−6^	1.02 × 10^−6^	47.5 ± 0.8	0.9979

* With an LaF_3_ single-crystal membrane (diameter of 8.0 mm and thickness of 1.0 mm, doped with 1.0% Eu); ** with an LaF_3_ single-crystal membrane (diameter of 8.0 mm and thickness of 1.5 mm, doped with 0.3% Eu); *** with an LaF_3_ single-crystal membrane (diameter of 8.0 mm and thickness of 5.0 mm, doped with 1.0% Eu); **** standard deviation (five replicates).

**Table 2:** The response characteristics of electrodes with Fe_x_O_y_ NPs.

Electrode	Internal Contact	Linear Response (mol/L)	Limit of Detection (mol/L) (LOD)	Slope (mV/dec) ± SD ****	*R* ^2^
4S *	solid	6.31 × 10^−6^	1.38 × 10^−6^	50.3 ± 0.8	0.9954
4E *	electrolyte	6.31 × 10^−6^	2.09 × 10^−6^	52.3 ± 1.1	0.9895
5S **	solid	1.58 × 10^−6^	5.01 × 10^−7^	62.4 ± 1.2	0.9887
5E **	electrolyte	1.58 × 10^−6^	2.88 × 10^−7^	52.8 ± 0.9	0.9969
6S ***	solid	6.31 × 10^−6^	2.40 × 10^−6^	59.5 ± 1.3	0.9839
6E ***	electrolyte	6.31 × 10^−6^	1.12 × 10^−6^	50.8 ± 0.6	0.9970

* With an LaF_3_ single-crystal membrane (diameter of 8.0 mm and thickness of 1.0 mm, doped with 1.0% Eu); ** with an LaF_3_ single-crystal membrane (diameter of 8.0 mm and thickness of 1.5 mm, doped with 0.3% Eu); *** with an LaF_3_ single-crystal membrane (diameter of 8.0 mm and thickness of 5.0 mm, doped with 1.0% Eu); **** standard deviation (nine replicates).

**Table 3:** The response characteristics of electrodes after washing the Fe_x_O_y_ NPs out from surface.

Electrode	Internal Contact	Linear Response (mol/L)	Limit of Detection (mol/L) (LOD)	Slope (mV/dec) ± SD ****	*R* ^2^
7S *	Solid	1.58 × 10^−6^	3.55 × 10^−7^	57.3 ± 0.4 *****	0.9932
7E *	Electrolyte	3.98 × 10^−7^	7.41 × 10^−8^	54.3 ± 0.5 *****	0.9969
8S **	Solid	1.58 × 10^−6^	3.31 × 10^−7^	52.9 ± 0.7	0.9954
8E **	Electrolyte	6.31 × 10^−6^	2.14 × 10^−6^	44.1 ± 1.2	0.9867
9S ***	Solid	6.31 × 10^−6^	1.38 × 10^−6^	54.8 ± 0.6	0.9945
9E ***	Electrolyte	1.58 × 10^−6^	5.24 × 10^−7^	50.8 ± 1.3	0.9875

* With an LaF_3_ single-crystal membrane (diameter of 8.0 mm and thickness of 1.0 mm, doped with 1.0% Eu); ** with an LaF_3_ single-crystal membrane (diameter of 8.0 mm and thickness of 1.5 mm, doped with 0.3% Eu); *** with an LaF_3_ single-crystal membrane (diameter of 8.0 mm and thickness of 5.0 mm, doped with 1.0% Eu); **** standard deviation (nine replicates); ***** standard deviation (23 replicates).

**Table 4:** Elemental analysis before Fe_x_O_y_ NP loading.

Element	Weight (%)	Atomic (%)
C	0.63	6.09
F	11.20	68.68
La	31.10	25.23
Totals	41.93	100.00

**Table 5:** Elemental analysis after Fe_x_O_y_ NP loading.

Element	Weight (%)	Atomic (%)
O	10.84	61.41
Na	0.25	0.98
Si	3.97	12.80
S	0.16	0.47
Cl	0.88	2.24
K	0.19	0.44
Fe	13.17	21.37
La	0.42	0.28
Total	29.87	99.99

## Supplementary Materials

Figure S1. Potentiometric response of internal solid contact LaF3 electrodes before Fe_x_O_y_ NP loading; Figure S2. Potentiometric response of internal electrolyte contact LaF3 electrodes before Fe_x_O_y_ NP loading, Figure S3. Potentiometric response of internal solid contact LaF3 electrodes after washing Fe_x_O_y_ NPs out; Figure S4. Potentiometric response of internal electrolyte contact LaF3 electrodes after washing Fe_x_O_y_ NPs out; Figure S5. Calibration curves for fluoride determination with 7E and commercial FISE.
