# Supplementary material for: Development of the New Fluoride Ion-Selective Electrode Modified with FexOy Nanoparticles
Source: Molecules. 2020 Nov 9;25(21):5213. doi: 10.3390/molecules25215213 (PMC7664936; doi:10.3390/molecules25215213)
Supplement: Supplementary file 1 [file molecules-25-05213-s001.pdf]

molecules

MDPI

Supplementary materials

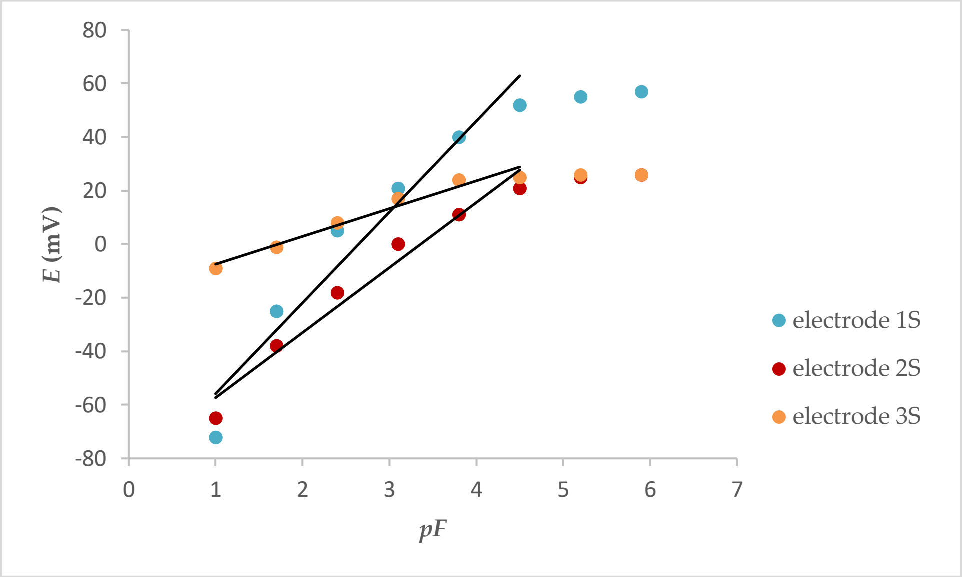

Figure S1. Potentiometric response of internal solid contact LaF_3 electrodes before Fe_xO_y NPs loading

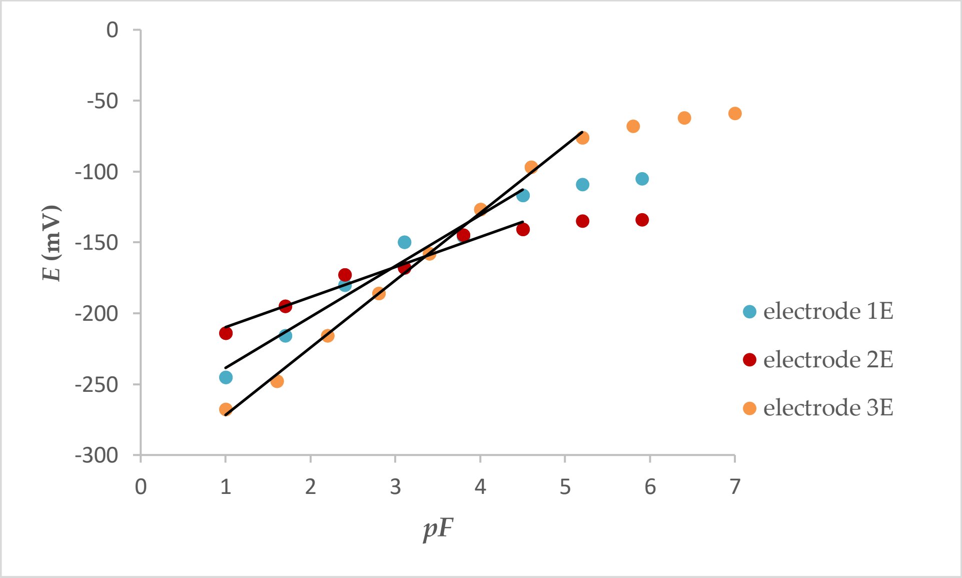

Figure S2. Potentiometric response of internal electrolyte contact LaF_3 electrodes before Fe_xO_y NPs loading

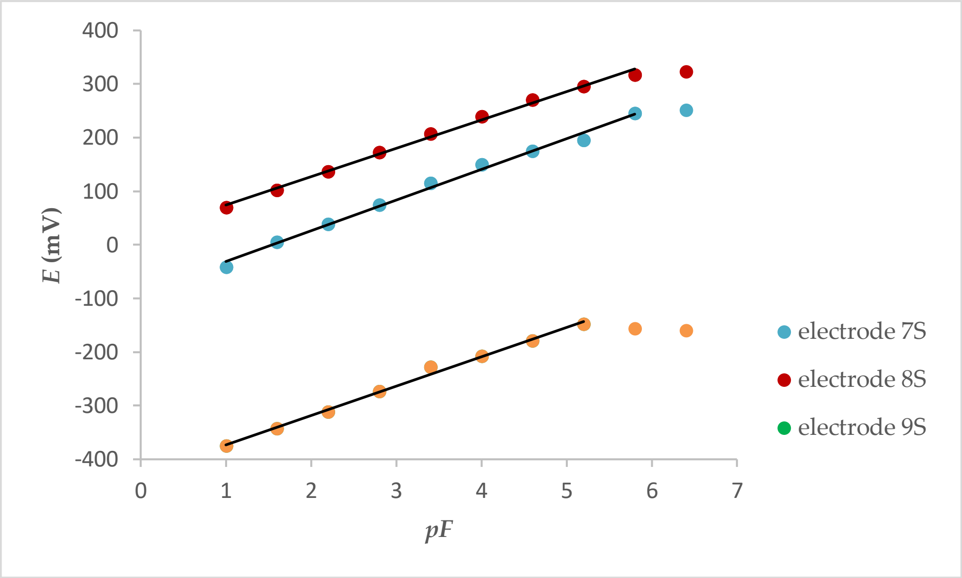

Figure S3. Potentiometric response of internal solid contact LaF_3 electrodes after washing Fe_xO_y NPs out

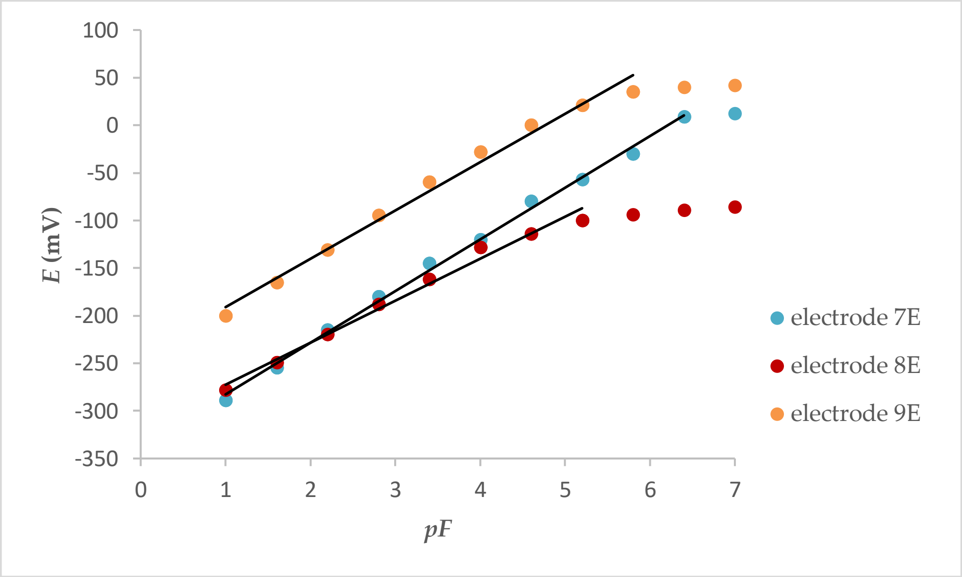

Figure S4. Potentiometric response of internal electrolyte contact LaF_3 electrodes after washing Fe_xO_y NPs out

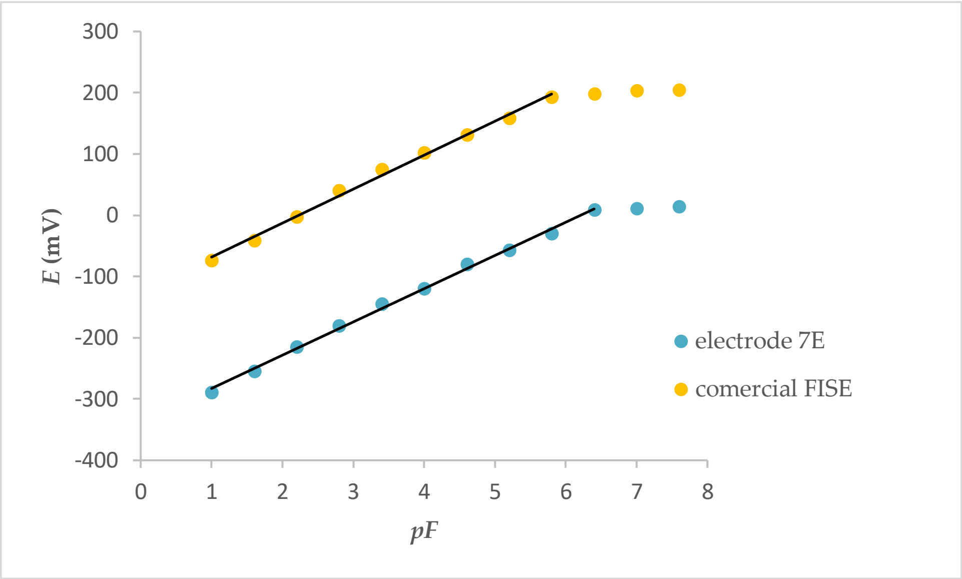

Figure S5. Calibration curves for fluoride determination with 7E and commercial FISE
